# Genome and proteome screening of *Onchocerca volvulus* reveal putative vaccine candidates

**DOI:** 10.1186/s43556-021-00062-z

**Published:** 2021-12-14

**Authors:** Abraham Sheyin, Thaddeus Terlumun Gbem, Daniel Danladi Gaiya, Jonathan Andrew Nok

**Affiliations:** 1 Department of Biochemistry, Ahmadu Bello University, Zaria, Nigeria; 2 Africa Centre of Excellence for Neglected Tropical Diseases and Forensic Biotechnology, Ahmadu Bello University, Zaria, Nigeria; 3 Department of Biology, Ahmadu Bello University, Zaria, Nigeria; 4 Air Force Institute of Technology, Nigerian Air Force Base, Kawo, Kaduna Nigeria

Dear Editor,

The global onchocerciasis burden is caused by the transmission of microfilariae into the skin of the human host via bites from infected female blackflies of the genius *Simulium*. Microfilariae gain access into the host system and migrate through the skin during blood meals causing severe dermatopathological conditions leading to visual impairment. The disease is also implicated in musculoskeletal pain, reduced body mass index and decreased work productivity. Onchocerciasis is a neglected tropical disease affecting more than 15 million people worldwide. Africa accounts for about 99% of active reported cases worldwide with recent records showing that over 172 million people predispose, require treatment for Onchocerciasis.

At present, there is no vaccine for the prevention of the disease and treatment is done by administering ivermectin that kills only the microfilaria, but does not kill the adult worm. The microfilaricidal action of ivermectin is achieved by consistent and repeated mass drug administration (MDA). This chemotherapeutic strategy was successful in Mali and Senegal with Columbia being verified as free of onchocercaisis by WHO in 2013. However, resistance to this drug is beginning to emerge in Africa and areas coendemic to loiasis. Recently, a study showed significant induced protective immunity in a mouse model by Ov-103, Ov-CPI-2 M and Ov-RAL-2 [1]. The feasibility of putative vaccine candidate targets (Ov-103 and Ov-RAL-2) is set for clinical and efficacy trials in humans. Recent studies depict transmembrane proteins potential targets for chimeric multi-epitope vaccine; stimulate cellular and humoral immune responses by inducing B-cells and IFN-γ based immunity [2]. Furthermore, reports focused on the use of single recombinant antigen did not generated sufficient immune responses for further clinical studies, also not exempting Ov-CP1-2 M homology in humans. A vaccine to prevent the infection is currently not available. There is need therefore to intensify the search for alternative and more effective treatment strategies. The availability of genome and proteome based databases as well as Bioinformatics tools have brought a deviation from the conventional approach to vaccine development.

In this study, a faster approach to vaccine discovery known as ‘reverse vaccinology’ was adopted [1]. Defined reverse vaccinology as the in silico screening of the total genome of a single pathogenic isolate or several isolates (the pan-genome) to find DNA sequences that encode proteins bearing vaccine targets. *Onchocerca volvulus* causes infection when a third-instar larva of its life cycle is transmitted to humans via the bite of the black fly, consequently resulting into onchocerciasis [2]. Using bioinformatics tools, we searched and identified putative vaccine targets of *Onchocerca volvulus*. The proteome of the pathogen screened was found to contain 802 proteins that are non-orthologues to human proteins which were used for further screening to isolate target proteins. TargetP predicted a total of 413 sequences to have a signal peptide. However, the cleavage sites predicted by SignalP for most protein sequences were one amino acid higher than the one predicted by TargetP (Table 1 and Supplementary Table 1). This is attributed to the fact that TargetP detects signal peptides at the N-terminal pre-sequence, whereas SignalP detects signal peptide cleavage sites at the C-terminal end of the pre-sequence [2].

**Table 1:** Uncharacterized protein sequences in *Onchocerca volvulus* sharing orthologues with *Dirofilaria immitis* and/or *Brugia malayi* and suggested putative functions

*O. volvulus* protein	Predicted function/description	*D. immitis* orthologues	*B. malayi* Orthologues	Single TMH	Secretory signal peptide	B-cell Epitopes	T-cell epitopes
A0A044RMM8	Impervious cuticlin protein	AF453385.1	XM 001898524.1	+	+	GVEGEPEIE, RNDEG, GGPTGQ, NG, SE, CSEP, GAATR	PTDLMAGQEAHVY
A0A044R5Q7	Impervious cuticlin protein	–	XM 001902142.1	+	+	DSPNG, QD, NIP, EFQTSPTTSTS, PDV, SGE, SPKTSET, STR, TV	STDDFILPQLTY
A0A044QSB0	Impervious cuticlin protein	–	XM 001895330.1	+	+	GVEGDPEI, CRSDEG, GPTGEPV, NG, GECARPECPEPQ, QPVQQPVE, DDNH	PTDLMAGQEAHVY
A0A044R521	Impervious cuticlin protein	–	XM 001902757.1	+	+	GVEGEPEIE, CRSDSG, GPTGT, DGKG, CPRPQCPE, PQ, AQ	LSDLMAGQEAHVY
A0A044VHR0	Zona pellucida-like domain containing protein	–	XM 001900794.1	+	+	FTP, QSM, RK, RR	VIDENGCTLDSY, LSSKHADFNANHEY, NSDATMHDY
A0A044RMF0	Hypothetical protein	–	XM 001896012.1	+	+	*	VSSDFSLFLY, VMDDVSSDFSLFLY, SSDFSLFLY
A0A044SEB3	Hypothetical protein	–	XM 001897289.1	+	+	TT, ESGK, VK, NR, ETS	SSSPAASTVIEMLY,
A0A044TJF8	Hypothetical protein	–	XM 001899259.1	+	+	NGA, IRPPP, SDSPSESAH, PRT, EKA	CTSWVQPQIGIY, TLDIRMTKTDRY, KTDRYDYLLQFCTY

Only tblastn results with E -value of 0.0 or E < 1e-50 was considered. ‘+’ = single transmembrane helice and secretory signal peptide present. B-cell epitopes are peptides with BepiPred score > 1.30. T-cell epitopes are peptides with the lowest percentile rank that can bind to Major Histocompatibility Complex I (HLA-A*01:01). ‘-’ = no orthologue, ‘*’ = less than 2 amino acids as epitope. *Onchocerca volvulus* proteins are referred to by their Uniprot sequence entry. *Dirofilaria immitis* and *Brugia malayi* orthologues are referred to by their accession number in National Center for Biotechnology Information (NCBI)

The program TMHMM predicted a total of 203 single TMH (Table 1). Proteins with more than one TMHs were not considered due to the high tendency for incorrect predictions of proteins with multiple transmembrane helices [2]. Phobius did not endorse any of the signal peptides and TMH predictions (made by SignalP, TargetP and TMHMM) due to both regions containing hydrophobic stretches as this confuses the programme and makes prediction difficult [2]. There was a consensus for common predictions among the four programs; SignalP, TargetP, TMHMM and Phobius for only 22 protein sequences (Supplementary Table 1) and immunogenic epitopes that can trigger humoral and cell-mediated immunity was predicted from these 22 sequences (Table 1 and Supplementary Table 2). A total of 29 epitopes of CD4+ T cells in the context of HLA-DRB1*01:01 and 40 epitopes of CD8+ T cells were predicted using the IEDB online tool in the context of the HLA-A*01:01 (Supplementary Table 3 and 4). In addition to the subtractive genomic approach, comparative genomics were also used. Fructose-1, 6-bisphosphate aldolase of *Dirofilaria immitis* has been reported [3] as a vaccine target. *Onchocerca volvulus* and *Dirofilaria immitis* share 97% sequence identity and a 98% sequence similarity with this protein. However, it was dropped because NCBI blastp (with refseq_ protein e as thdatabase name) alignment output showed that it had > 60% identical to four fructose-1, 6-bisphosphate aldolase of humans (Supplementary Fig. 1). This discovery casts doubt on the position of [4] that fructose-1, 6-bisphosphate aldolase of *Onchocerca volvulus* can serve as a potential vaccine candidate. Similarly, chitinase of *Onchocerca volvulus* and *Brugia malayi* had a sequence identity of 87% and a sequence similarity of 77%. Further, both sequence alignments had an E value of 0.0, i.e., < 1e-179, a significant match in each case showing proof of homology [4]. Our inclusion of chitinase in the vaccine candidate list coincides with an earlier position of [5] that chitinase of *Onchocerca volvulus* has some vaccine potential [5]. The possible function/description of some of the uncharacterized proteins predicted to be putative vaccine candidates is given in Table 1.

This study has shown reverse vaccinology as a useful tool in screening and identification of antigenic proteins; without culturing any protein of the pathogen we were able to identify 23 putative vaccine candidates, from which many epitopes were predicted and we recommend that these candidates be used in *Onchocerca volvulus* experimental vaccine research, especially, the ones found to be orthologues to *Brugia malayi/Dirofilaria immitis* since there are animal models for the duo, but none for *Onchocerca volvulus*. In order to avoid the risk of autoimmunity, Fructose-1,6-bisphosphate aldolase of *Onchocerca volvulus* cannot serve as a vaccine candidate as it was found to be an orthologue of humans. Also, possible functions and description of some of the uncharacterized proteins have been suggested and their validation is necessary.

## Supplementary Information

**Additional file 1: Supplementary Table 1**. Uncharacterized protein sequences of *Onchocerca volvulus* found to be putative vaccine candidates which are non-orthologues to *Dirofilaria immitis* and *Brugia malayi*. **Supplementary Table 2**. Linear B-cell epitopes of *Onchocerca volvulus.* **Supplementary Table 3**. Peptides of *O. volvulus* predicted to bind to MHC IHLA-A*01:01 allele. **Supplementary Table 4**. Peptides of *O. volvulus* predicted to bind to MHC II HLA-DRB1*01:01 allele. **Supplementary Table 5**. Possible function of some of the 22 putative vaccine candidates. **Fig. 1**. National Centre for Biotechnology Information (NCBI) BLAST output showing alignment of query (established potential vaccine candidate- Fructose bisphosphate aldolase of *Dirofilaria immitis*) with subject (Fructose − 1,6- bisphosphate aldolase of *Onchocerca volvulus*) and their alignment details.

## Data Availability

The datasets generated and/ or analyzed during the current study are available from the corresponding author upon reasonable request.

## Abbreviations

HLA-A: Human Leukocyte Antigen
NCBI: National Center for Biotechnology Information
MHC: Major Histocompatibility Complex
TMHs: Transmembrane Helices
TMH: Transmembrane Helix
TMHMM: Tied Mixture Hidden Markov Model
