# Supplementary material for: Genome and proteome screening of Onchocerca volvulus reveal putative vaccine candidates
Source: Mol Biomed. 2021 Dec 14;2:39. doi: 10.1186/s43556-021-00062-z (PMC8670411; doi:10.1186/s43556-021-00062-z)
Supplement: Supplementary file 1 — Additional file 1: Supplementary Table 1. Uncharacterized protein sequences of Onchocerca volvulus found to be putative vaccine candidates which are non-orthologues to Dirofilaria immitis and Brugia malayi. Supplementary Table 2. Linear B-cell epitopes of Onchocerca volvulus. Supplementary Table 3. Peptides of O. volvulus predicted to bind to MHC IHLA-A*01:01 allele. Supplementary Table 4. Peptides of O. volvulus predicted to bind to MHC II HLA-DRB1*01:01 allele. Supplementary Table 5. Possible function of some of the 22 putative vaccine candidates. Fig. 1. National Centre for Biotechnology Information (NCBI) BLAST output showing alignment of query (established potential vaccine candidate- Fructose bisphosphate aldolase of Dirofilaria immitis) with subject (Fructose − 1,6- bisphosphate aldolase of Onchocerca volvulus) and their alignment details. [file 43556_2021_62_MOESM1_ESM.docx]

**Supplementary Table 1** Uncharacterized protein sequences of *Onchocerca volvulus* found to be putative vaccine candidates which are non-orthologues to *Dirofilaria immitis* and *Brugia malayi*.

| *Onchocerca volvulus protein* | Single TMH | Secretory peptide | B-cell epitopes | T-cell epitopes |
| --- | --- | --- | --- | --- |
| A0A044TMU6 | + | + | * | MMCPQNSLTYYY, FVSLFLFGY |
| A0A044TM82 | + | + | NEGD, SSPDAR, GSGY, DNGPNPDD, TD | ITDQRGYFF |
| A0A044U293 | + | + | QPSSWPNDSTTDH, DSSESETNPE, SDQTN | ETDTEEINSSHMY |
| A0A044V9Z2 | + | + | PG, GNESEKK, NTTATPEKEYS | NTTATPEKEY |
| A0A044RIX6 | + | + | * | CSAINPHGY |
| A0A044SF87 | + | + | DPL | ETEHLNGLSLPNEY |
| A0A044SCX7 | + | + | NP, RI | STNDHRLYY |
| A0A044UWG2 | + | + | * | PASSIFHYY |
| A0A044VAP0 | + | + | EYESSGD, KNAN, NFD | RTSTPSLTPIAY |
| A0A044TBY3 | + | + | * | CSKESDSCY |
| A0A044UNM4 | + | + | TCKG | FSSSSPFQCY |
| A0A044RG47 | + | + | SEVPEST | NTSAIHEGIY |
| A0A044TDN4 | + | + | * | LTDQIAEKAKQTVV |
| A0A044V162 | + | + | EP | LTTMCILQWIFNRY |

'+' = single transmembrane helice and secretory signal peptide present. B-cell epitopes are peptides with score > 1.30. T-cell epitopes are peptides with lowest percentile rank that can bind to Major Histocompatibility Complex I (HLA-A*01:01). ‘*’ ≥ 2 amino acids as epitope. *Onchocerca volvulus* proteins are referred to by their Uniprot sequence entry.

**Supplementary Table 2** Linear B-cell epitopes of *Onchocerca volvulus*

| **Sequence entry** | **Epitope (score 1.30)** |
| --- | --- |
| A0A044TMU6 | - |
| A0A044TM82 | NEGD, SSPDAR, GSGY, DNGPNPDD, TD |
| A0A044U293 | QPSSWPNDSTTDH, DSSESETNPE, SDQTN |
| A0A044V9Z2 | PG, GNESEKK, NTTATPEKEYS |
| A0A044RIX6 | - |
| A0A044SF87 | DPL |
| A0A044SCX7 | NP, RI |
| A0A044UWG2 | - |
| A0A044R5Q7 | DSPNG, QD, NIP, EFQTSPTTSTS, PDV, SGE, SPKTSET, STR, TV |
| A0A044VAP0 | EYESSGD, KNAN, NFD |
| A0A044VHR0 | FTP, QSM, RK, RR |
| A0A044TBY3 | - |
| A0A044UNM4 | TCKG |
| A0A044RG47 | SEVPEST |
| A0A044RMF0 | - |
| A0A044TDN4 | - |
| A0A044RMM8 | GVEGEPEIE, RNDEG, GGPTGQ, NG, SE, CSEP, GAATR |
| A0A044QSB0 | GVEGDPEI, CRSDEG, GPTGEPV, NG, GECARPECPEPQ, QPVQQPVE, DDNH |
| A0A044SEB3 | TT, ESGK, VK, NR, ETS |
| A0A044R521 | GVEGEPEIE, CRSDSG, GPTGT, DGKG, CPRPQCPE, PQ, AQ |
| A0A044TJF8 | NGA, IRPPP, SDSPSESAH, PRT, EKA |

The total of 69 linear B-cell epitopes predicted from the 22 sequences earlier screened for MHC I and MHC II peptide binding are presented in Table 1. Their lengths are from 1 – 13 amino acid residues long, but epitopes with length less than two were not considered. The epitopes contain a few Tyrosine (Y) residues and many Arginine (R), Glycine (G) and Serine (S) residues.

**Supplementary Table** **3** Peptides of *O. volvulus* predicted to bind to MHC IHLA-A*01:01 allele

| **Rank** | **Sequence Entry** | **Peptide** | **Length** | **Start-End** | **Percentile rank** |
| --- | --- | --- | --- | --- | --- |
| 1 | A0A044TMU6 | MMCPQNSLTYYY  FVSLFLFGY | 12  9 | 54-65  8-16 | 0.4  0.4 |
| 2 | A0A044TM82 | ITDQRGYFF | 9 | 64-72 | 0.4 |
| 3 | A0A044U293 | ETDTEEINSSHMY | 13 | 58-70 | 0.1 |
| 4 | A0A044V9Z2 | NTTATPEKEY | 10 | 147-156 | 0.5 |
| 5 | A0A044RIX6 | CSAINPHGY | 9 | 91-99 | 0.25 |
| 6 | A0A044SF87 | ETEHLNGLSLPNEY | 14 | 122-135 | 0.2 |
| 7 | A0A044SCX7 | STNDHRLYY | 9 | 122-130 | 0.2 |
| 8 | A0A044UWG2 | PASSIFHYY | 9 | 48-56 | 0.45 |
| 9 | A0A044R5Q7 | STDDFILPQLTY | 12 | 213-224 | 0.1 |
| 10 | A0A044VAP0 | RTSTPSLTPIAY | 12 | 324-335 | 0.2 |
| 11 | A0A044VHR0 | VIDENGCTLDSY  LSSKHADFNANHEY  NSDATMHDY | 12  14  9 | 343-354  158-171  140-148 | 0.2  0.2  0.2 |
| 12 | A0A044TBY3 | CSKESDSCY | 9 | 43-51 | 0.7 |
| 13 | A0A044UNM4 | FSSSSPFQCY | 10 | 15-24 | 0.2 |
| 14 | A0A044RG47 | NTSAIHEGIY | 10 | 104-113 | 0.3 |
| 15 | A0A044RMF0 | VSSDFSLFLY  VMDDVSSDFSLFLY  SSDFSLFLY | 10  14  9 | 428-437  424-437  429-437 | 0.2  0.2  0.2 |
| 16 | A0A044TDN4 | LTDQIAEKAKQTVV | 14 | 38-51 | 0.4 |
| 17 | A0A044V162 | LTTMCILQWIFNRY | 14 | 219-232 | 0.2 |
| 18 | A0A044RMM8 | PTDLMAGQEAHVY | 13 | 223-235 | 0.1 |
| 19 | A0A044QSB0 | PTDLMAGQEAHVY | 13 | 230-242 | 0.1 |
| 20 | A0A044SEB3 | SSSPAASTVIEMLY | 14 | 396-409 | 0.2 |
| 21 | A0A044R521 | LSDLMAGQEAHVY | 13 | 233-245 | 0.1 |
| 22 | A0A044TJF8 | CTSWVQPQIGIY  TLDIRMTKTDRY  KTDRYDYLLQFCTY | 12  12  14 | 21-32  58-69  65-78 | 0.2  0.2  0.2 |

The lowest and highest percentile rank is 0.1 and 0.7 respectively. Rank 22, 15 and 11 have three peptides each that would theoretically bind to the allele. Rank 1 has two peptides that would bind to the allele and the remaining 18 sequence entries have one peptide each that would bind to

36 the allele. The lengths of the peptides are 9-14 residues long.

**Supplementary Table** 4 Peptides of *O. volvulus* predicted to bind to MHC II HLA-DRB1*01:01 allele

| **Rank** | **Sequence Entry** | **Peptide** | **Length** | **Start-End** | **Percentile rank** |
| --- | --- | --- | --- | --- | --- |
| 1 | A0A044TMU6 | LFLFGYLNVNSAQAM  FLFGYLNVNSAQAMN | 15  15 | 11-25  12-26 | 3.95  3.95 |
| 2 | A0A044TM82 | LWWFLPNAANALFAL | 15 | 8-22 | 0.09 |
| 3 | A0A044U293 | LLFILLFCNMLLARL | 15 | 7-21 | 0.77 |
| 4 | A0A044V9Z2 | TILIIIVASAIVLYT | 15 | 173-187 | 0.60 |
| 5 | A0A044RIX6 | VLTSFVTLVTLIIMR  LTSFVTLVTLIIMRF | 15  15 | 236-250  237-251 | 0.96  0.96 |
| 6 | A0A044SF87 | SAILFTALIVTFTLL | 15 | 75-89 | 3.24 |
| 7 | A0A044SCX7 | LAYFMIILLIVLHLL | 15 | 200-214 | 2.51 |
| 8 | A0A044UWG2 | TWVIVVLAVIAVMML | 15 | 72-86 | 2.05 |
| 9 | A0A044R5Q7 | LLSMWILSGLSLVSI | 15 | 512-526 | 2.91 |
| 10 | A0A044VAP0 | MKILISLAIIIAARL  KILISLAIIIAARLH  LISLAIIIAARLHSI | 15  15  15 | 1-15  2-16  4-18 | 0.77  0.77  0.77 |
| 11 | A0A044VHR0 | QSPFKGIHAMLVRNC | 15 | 317-331 | 2.18 |
| 12 | A0A044TBY3 | MIILLTFFLLHASLL  IILLTFFLLHASLLW  ILLTFFLLHASLLWN  LLTFFLLHASLLWNV  LTFFLLHASLLWNVV | 15  15  15  15  15 | 1-15  2-16  3-17  4-18  5-19 | 0.04  0.04  0.04  0.04  0.04 |
| 13 | A0A044UNM4 | ITNIHLFISNLFITF | 15 | 114-128 | 5.88 |
| 14 | A0A044RG47 | QFFASILSSLFIAVL | 15 | 140-154 | 0.77 |
| 15 | A0A044RMF0 | KARLIVIASARDILV | 15 | 252-266 | 0.62 |
| 16 | A0A044TDN4 | ILILILLKMLWSGLV  LILILLKMLWSGLVW  LILLKMLWSGLVWCI | 15  15  15 | 101-115  102-116  104-118 | 2.05  2.05  2.05 |
| 17 | A0A044V162 | TIVSLLFVIWSPAIK  IVSLLFVIWSPAIKT  VSLLFVIWSPAIKTA  SLLFVIWSPAIKTAQ  LLFVIWSPAIKTAQV | 15  15  15  15  15 | 4-18  5-19  6-20  7-21  8-22 | 5.46  5.46  5.46  5.46  5.46 |
| 18 | A0A044RMM8 | FGFSMFMGLSIALIA  GFSMFMGLSIALIAA  FSMFMGLSIALIAAV  SMFMGLSIALIAAVI | 15  15  15  15 | 352-366  353-367  354-368  355-369 | 0.42  0.42  0.42  0.42 |
| 19 | A0A044QSB0 | CFSNGTFAILIAMFG | 15 | 363-377 | 2.51 |
| 20 | A0A044SEB3 | VLGFFMLLLGGAIGV  LGFFMLLLGGAIGVG | 15  15 | 613-627  614-628 | 0.03  0.03 |
| 21 | A0A044R521 | ILLIILFGAMLAIGS | 15 | 363-377 | 0.28 |
| 22 | A0A044TJF8 | IWFLIFLAFILLLCL | 15 | 185-199 | 1.15 |

The lowest and highest percentile rank observed for the peptides predicted are 0.03 and 5.88 respectively. Rank 12 and 17 have five peptides each that can bind to the allele. Rank 18 has four peptides, rank 16 and 10 have three each. Rank 1, 5 and 20 have two peptides each and the remaining 14 sequences have one peptide that can bind to the allele.

**Supplementary Table 5** Possible function of some of the 22 putative vaccine candidates

| ***Onchocerca volvulus* protein** | **Predicted description** | | ***Dirofilaria immitis* orthologue** | |  | |  | | ***Brugia malayi* orthologue** | |  | |  | |
| --- | --- | --- | --- | --- | --- | --- | --- | --- | --- | --- | --- | --- | --- | --- |
|  |  | Acession no | | E-value | | % Identity | | Acession no | | E-value | | % Identity | |  |
| A0A044RMM8 | Impervious cuticlin protein | AF453385.1 | | 0.0 | | 62 | | XM 001898524.1 | | 0.0 | | 90 | |  |
| A0A044R5Q7 | Impervious cuticlin protein | - | | - | | - | | XM 001902142.1 | | 1.E-65 | | 88 | |  |
| A0A044QSB0 | Impervious cuticlin protein | - | | - | | - | | XM 001895330.1 | | 0.0 | | 73 | |  |
| A0A044R521 | Impervious cuticlin protein | - | | - | | - | | XM 001902757.1 | | 0.0 | | 77 | |  |
| A0A044VHR0 | Zona pellucida-like domain containing protein | - | | - | | - | | XM 001900794.1 | | 0.0 | | 62 | |  |
| A0A044RMF0 | Hypothetical protein | - | | - | | - | | XM 001896012.1 | | 0.0 | | 60 | |  |
| A0A044SEB3 | Hypothetical protein | - | | - | | - | | XM 001897289.1 | | 0.0 | | 68 | |  |
| A0A044TJF8 | Hypothetical protein | - | | - | | - | | XM 001899259.1 | | 1E-138 | | 55 | |  |

Only tblastn E -value of 0.0 or E < 1e-50 was considered. '-' = no orthologue. *O. volvulus* proteins are referred to by their Uniprot sequence entry. *Dirofilaria immitis* and *Brugia malayi* orthologues are referred to by their accession number in National center for Biotechnology information (NCBI). Expectation value (E value) and % identity are NCBI blast output parameters.

**Figure 1** National Centre for Biotechnology Information (NCBI) BLAST output showing alignment of query (established potential vaccine candidate- Fructose bisphosphate aldolase of *Dirofilaria immitis*) with subject (Fructose -1,6- bisphosphate aldolase of *Onchocerca volvulus*) and their alignment details.

**Material and methods**

**Sequence retrieval and proteome analysis**

The complete 4551uncharacterized protein sequences of *Onchocerca volvulus* were retrieved from UniProt (http://www.uniprot.org/) in FASTA format. To avoid autoimmunity possibilities, 802 proteins, which are highly non-orthologues to human proteins were identified and used for subsequent screening ([www.ncbi.nlm.nih.gov](http://www.ncbi.nlm.nih.gov) ). The proteins were then subjected to signal peptide analyses using SignalP 4.1 version and TargetP 1.1 version ([www.cbs.dtu.dk/services/SignalP/](http://www.cbs.dtu.dk/services/SignalP/) and [www.cbs.dtu.dk/services/TargetP](http://www.cbs.dtu.dk/services/TargetP)) to discriminate classical secretory and non-secretory proteins. SignalP (version 4.1) which predicts the presence and location of signal peptidase cleavage site in the C-terminal end of the pre-sequence, TargetP (version 1.1) which predicts the presence and length of signal peptides in the N-terminal pre-sequences. As at the time of the research, we had access to some old versions of the prediction software packages.

**Prediction of transmembrane helice (TMH)**

Phobius (version 1.01) a combined signal peptide and TMH predictor was used to eliminate false positives, discriminates between Transmembrane helices and Secretory peptides thereby adding endorsement to the predictions (since signal peptides and TMH both contain hydrophobic amino acids that may confuse predictions).The program TMHMM was used to predict TMHs (<http://www.cbs.dtu.dk/services/TMHMM-2.0/>).

**T-cell epitopes and B-cell epitopes**

The key objective of epitope prediction is to design a molecule that can replace an antigen in the process of either antibody production or antibody detection. The 22 protein sequences predicted by SignalP, TargetP, TMHMM and Phobius to possess both signal peptide and transmembrane helice were further screened for T-cell epitopes binders (MCH I and MCH II). T lymphocytes are divided into two groups: CD8+ and CD4+ T cells. CD4+ T cells are the essential component of protective immunity. We therefore analyzed the epitopes of CD4+ T cells in the context of HLA-DRB1*01:01. Next, epitopes of CD8+ T cells were predicted using the IEDB online tool in the context of the HLA-A*01:01. Screening for MHC I (HLA-A*01:01) was done using the Immune Epitope database (IEDB) analysis resource consensus tool (<http://tools.iedb.org/mhci/>) which combines predictions from Artificial Neural Network, ANN aka NetMHC, SMM as well as Comblib. T-cells binding to MHC II predictions were also made using the same tool, IEDB that combines the methods. A total of 69 linear B-cell epitopes predicted from the 22 sequences earlier screened for MHC I and MHC II peptide binding using BepiPred 1.0 (http://www.cbs.dtu.dk/services/BepiPred/) the score threshold for epitope assignment used was 1.30.

**Determining orthologues of *Dirofilaria immitis* and *Brugia malayi***

Fructose bisphosphate aldolase, an established potential vaccine candidate of *D. immitis* and chitinase*,* an established potential vaccine candidate of *B. malayi* were used as queries against the *O. volvulus* genome to find orthologues using the NCBI program tBLASTn with NCBI default settings.
